# Within-host co-evolution of KPC variants: plasmid-mediated dissemination of *bla*_Kpc-194_ and *bla*_Kpc-33_ in ST11-KL64 hypervirulent *Klebsiella pneumoniae* driving ceftazidime-avibactam resistance

**DOI:** 10.1128/spectrum.03257-25

**Published:** 2026-03-16

**Authors:** Li Ding, Xiangbing Wu, Qiang Xie, Liting Liu, Bingshao Liang, Siquan Shen, Yan Guo, Jing Chen, Fupin Hu

**Affiliations:** 1 Institute of Antibiotics, Huashan Hospital, Fudan University 198171, Shanghai, China; 2 Key Laboratory of Clinical Pharmacology of Antibiotics, Ministry of Health 223520, Shanghai, China; 3 Department of Laboratory Medicine, Wenzhou Central Hospital, Wenzhou, Zhejiang, China; 4 Department of Laboratory Medicine, The Affiliated Chuzhou Hospital of Anhui Medical University (The First People's Hospital of Chuzhou) 612138, Chuzhou, Anhui, China; 5 Zhongshan Hospital of Traditional Chinese Medicine 612138, Zhongshan, Guangdong, China; 6 Clinical Laboratory, Guangzhou Women and Children's Medical Center, Guangzhou Medical University, Guangzhou, China; 7 Hangzhou Matridx Biotechnology Co., Ltd, Zhejiang, China; Zhejiang University, Hangzhou, China

**Keywords:** KPC-194, KPC-2, *K. pneumoniae*, ceftazidime-avibactam, KPC variants

## Abstract

KPC variants are the primary cause of treatment failure in patients with *Klebsiella pneumoniae* infections. This study reports the molecular mechanism by which two novel KPC variants (KPC-194 and KPC-33), isolated from a single patient, mediate resistance to ceftazidime-avibactam in ST11-KL64 *K. pneumoniae*, as well as the evolutionary trajectory of these variants within the host. The broth microdilution method (BMD) was used to determine bacterial susceptibility to antimicrobial agents. Whole-genome sequencing (WGS) technology was employed to identify the drug-resistant genes, virulence genes, and genetic environment carried by the bacterial strains. Molecular cloning experiments and plasmid conjugation experiments were conducted to clarify the susceptibility of KPC-194 to ceftazidime-avibactam and carbapenem. The BMD showed that the KPC-194-producing *K. pneumoniae* strain was resistant to ceftazidime-avibactam and other antimicrobial agents but susceptible to imipenem (with a minimum inhibitory concentration [MIC] of 0.5 mg/L). Compared with KPC-2, KPC-194 had two amino acid changes, namely, D179Y and P183L. In comparison with *Escherichia coli* EC 600, the MIC of ceftazidime-avibactam against *E. coli* EC 600 carrying the *bla*_KPC-194_ plasmid increased by 256-fold. When compared with pHSG398-DH5α, the MIC of ceftazidime-avibactam against the cloned strain *bla*_KPC-194_-pHSG398-DH5α was elevated by 64-fold. WGS revealed that *bla*_KPC-194_ was located on both the IncFII(pHN7A8)-type plasmid and the IncR-type plasmid and that it was horizontally transferred from the IncR-type plasmid to the IncFII(pHN7A8)-type plasmid via an IS*26*-mediated replicative transposition mechanism. This study elucidates the key mechanism by which the novel KPC variant, KPC-194 (D179Y/P183L), mediates resistance to ceftazidime-avibactam.

**IMPORTANCE:**

This study elucidates the critical molecular mechanism and evolutionary pathway of a novel KPC variant, KPC-194, that confers resistance to the last-resort antibiotic combination ceftazidime-avibactam in a high-risk *Klebsiella pneumoniae* strain. We identified that two amino acid substitutions (D179Y/P183L) in KPC-194 are responsible for ceftazidime-avibactam resistance. Crucially, our work reveals a dual-threat dynamic: the resistance phenotype is not only caused by the KPC mutation but also profoundly exacerbated by horizontal gene transfer. *bla*_KPC-194_ mobilized from a low-risk IncR plasmid to a highly transmissible IncFII plasmid via IS26-mediated replicative transposition. This event dramatically enhances the potential for widespread dissemination among clinical pathogens.

## INTRODUCTION

*Enterobacterales* are among the most common pathogens causing hospital- and community-acquired infections, which can lead to infections in various organs and tissues, such as the respiratory system, urinary system, and hematological system. Carbapenems are key drugs for the treatment of drug-resistant gram-negative bacilli and play a crucial role in clinical practice. However, in recent years, with the global prevalence of carbapenem-resistant *Enterobacterales* (CRE), especially carbapenem-resistant *Klebsiella pneumoniae* (CRKP), they have posed a significant threat to global public health (1, 2). Based on this, the World Health Organization updated its list of drug-resistant bacteria in May 2024, and CRE remains classified as a “critical priority threat.” There is an urgent need to develop new antimicrobial agents and implement preventive measures to combat the global spread of drug-resistant bacteria.

Producing carbapenemases is the primary mechanism underlying carbapenem resistance in *K. pneumoniae*, including class A serine carbapenemases (e.g., KPC-2 and KPC-3), class B metallo-carbapenemases (e.g., NDM, IMP, and VIM), and class D OXA-type carbapenemases (e.g., OXA-48-like carbapenemase) (3). The study has shown that 92% of clinically isolated carbapenemase-producing CRKP strains in South America produce KPC enzymes, with KPC-2 and KPC-3 accounting for a comparable proportion (4). In contrast, 96% of carbapenemase-producing CRKP strains in China produce KPC-2 (5). Ceftazidime-avibactam is a novel β-lactam/β-lactamase inhibitor combination. Since its approval for marketing in 2015, it has become a key drug for the treatment of CRKP strains producing KPC-2/KPC-3 carbapenemases (6, 7). The 2024 guidelines of the Infectious Diseases Society of America recommend ceftazidime-avibactam as one of the first-line antimicrobial agents for treating non-simple cystitis caused by CRKP that do not produce MBLs (6). However, with the widespread clinical use of ceftazidime-avibactam, cases have been reported where strains undergo KPC mutations during treatment, which mediate bacterial resistance to ceftazidime-avibactam and ultimately lead to treatment failure. This poses new challenges to clinical anti-infective therapy. In recent years, clinical reports on KPC variants have shown an outbreak-like increasing trend, and the National Center for Biotechnology Information (NCBI) database has included more than 250 KPC variants (https://www.ncbi.nlm.nih.gov/pathogens/refgene/#KPC) (8, 9).

In this study, we report a novel KPC variant, *bla*_KPC-194_, which confers resistance to ceftazidime-avibactam in *K. pneumoniae*. While *bla*_KPC-194_ was identified from a blood culture isolate, the *bla*_KPC-33_ variant was concurrently detected in sputum from the same patient during the same period. We characterized the changes in bacterial resistance mediated by KPC variants through molecular cloning assays and plasmid conjugation assays and clarified the transmission mechanism of the *bla*_KPC_ using sequencing technology.

## MATERIALS AND METHODS

### Clinical strains

In this study, three *K. pneumoniae* strains were isolated from a 94-year-old male patient in a tertiary first-class hospital in Wenzhou, Zhejiang Province. Among them, KPN001 was a ceftazidime-avibactam-susceptible *K. pneumoniae* strain, while KPN002 and KPN003 were ceftazidime-avibactam-resistant *K. pneumoniae* strains. All strains were identified using matrix-assisted laser desorption/ionization time-of-flight mass spectrometry (bioMérieux, France).

### Antimicrobial susceptibility testing

In accordance with the standards of CLSI M100, the broth microdilution method (BMD) was used to determine the minimum inhibitory concentration (MIC) of antimicrobial agents against the study strains (10). For cefiderocol and imipenem-relebactam, the E-test method was employed for MIC detection. Except for tigecycline, eravacycline, and aztreonam-avibactam, the antimicrobial susceptibility testing (AST) results of all other agents were interpreted in accordance with the 2024 edition of the CLSI document. Tigecycline (S: ≤0.5 mg/L, R: >0.5 mg/L) and eravacycline (S: ≤1 mg/L) were interpreted using the breakpoint standards of EUCAST and ECAST, respectively. Aztreonam-avibactam was interpreted in accordance with the EUCAST breakpoint standards (https://www.eucast.org/bacteria/clinical-breakpoints-and-interpretation/clinical-breakpoint-tables/). The quality control strains used for susceptibility testing were *Escherichia coli* ATCC 25922 and *K. pneumoniae* BAA 1705 (*bla*_KPC-2_ positive).

### Detection of carbapenemase types

The APB/EDTA method was used for screening carbapenemase types in CRKP, with specific procedures adapted from previous studies (11). Meanwhile, the NG-Test Carba-5 was used to detect the specific types of carbapenemases (KPC, NDM, VIM, IMP, and OXA-48) carried by the bacteria, in accordance with the manufacturer’s instructions.

### Whole-genome sequencing and analysis

Genomic DNA of the isolates was extracted using a Qiagen commercial kit according to the manufacturer’s recommendations. Sequencing was performed using the Illumina MiSeq platform (Illumina Inc.) with a paired-end approach (2 × 300 bp), and complete chromosomal and plasmid sequences were obtained using the MinION platform (Nanopore, Oxford, UK). Plasmids were annotated and aligned using the Proksee website (proksee.ca). The sequence type, serotype, capsular type, and other types of *K. pneumoniae* were analyzed using the Pathogen online website (pathogen.watch). Genes were annotated using the RAST website (ast.nmpdr.org/rast.cgi), and the genetic environment around the genes was mapped using Easyfig 2.2.5 (12).

### Plasmid conjugation assay

Plasmid conjugation assays were performed to investigate the characteristics of different *bla*_KPC_-carrying plasmids, following the method described by Huang et al. (13). Briefly, plasmids from the donor strains were transferred to the recipient strain, rifampicin-resistant *E. coli* EC600, through conjugation. Target bacteria were screened on Mueller–Hinton (MH) agar containing rifampicin (50 mg/L) and ampicillin (50 mg/L). The *bla*_KPC_ and relevant resistance genes (including *rmtB*, *iutA*, *qnrS1*, and *bla*_CTX-M-65_) were verified by PCR and Sanger sequencing, aimed at distinguishing which plasmid was transferred to the recipient strain. The experiment was conducted in triplicate.

### Cloning assay of KPC variants

Molecular cloning assays were performed to investigate whether KPC variants contribute to the resistance of clinical strains to ceftazidime-avibactam, following the method described by Shen et al. (13). Briefly, the full-length *bla*_KPC_ fragment was amplified from the original strain using KPC primers (forward primer, CCATGATTACGAATTGTGCGCGGAACCCCTATTTG; reverse primer, CGACTCTAGAGGATCCAATAGATGATTTTCAGAGCCTTAC) and PCR amplification. The PCR products were then purified using a MolPure PCR purification kit (Yeasen, China). The pHSG398 plasmid DNA was digested with two FastDigest restriction enzymes (BamHI and EcoRI, purchased from Thermo Fisher Scientific Inc.) at 37°C for 15 minutes, followed by enzyme inactivation at 80°C for 5 minutes. The cloning reaction, which involved joining the KPC insert to the linearized plasmid, was performed using the Hieff Clone Plus One Step Cloning Kit (Yeasen) according to the manufacturer’s protocol. The target bacteria were screened on MH agar containing chloramphenicol (50 mg/L) and ampicillin (50 mg/L), and the presence of the *bla*_KPC_ gene was verified by PCR and Sanger sequencing. DH5α containing *bla*_KPC-194_-pHSG398, DH5α containing *bla*_KPC-33_-pHSG398, and DH5α containing *bla*_KPC-2_-pHSG398 were successfully obtained. AST was then performed on the resulting clones.

## RESULTS

### Clinical information

**Fig 1:**
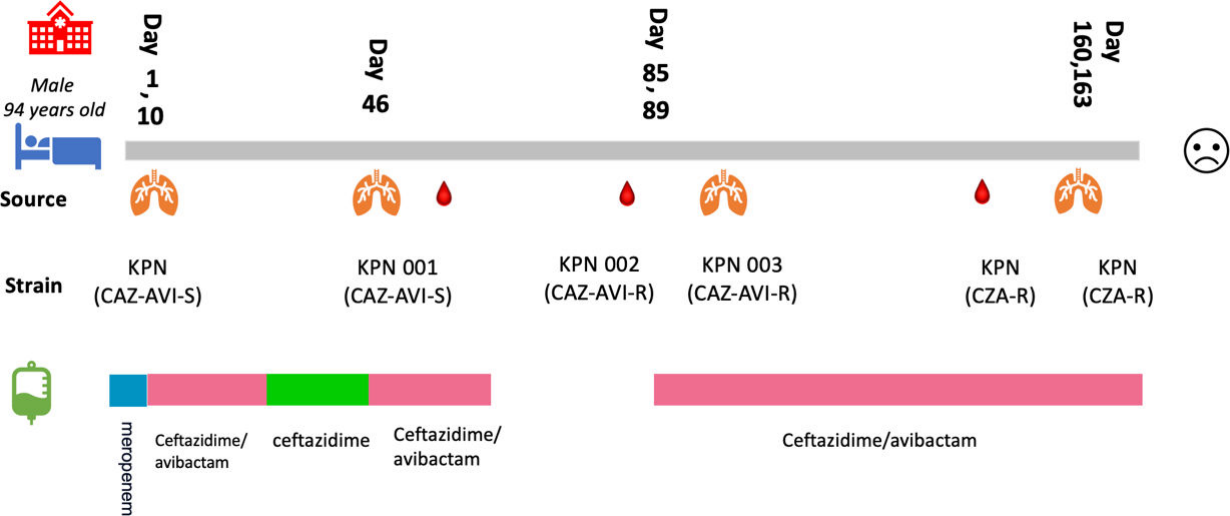
Bacterial culture results and antimicrobial treatment information of the patient.

**Fig 2:**
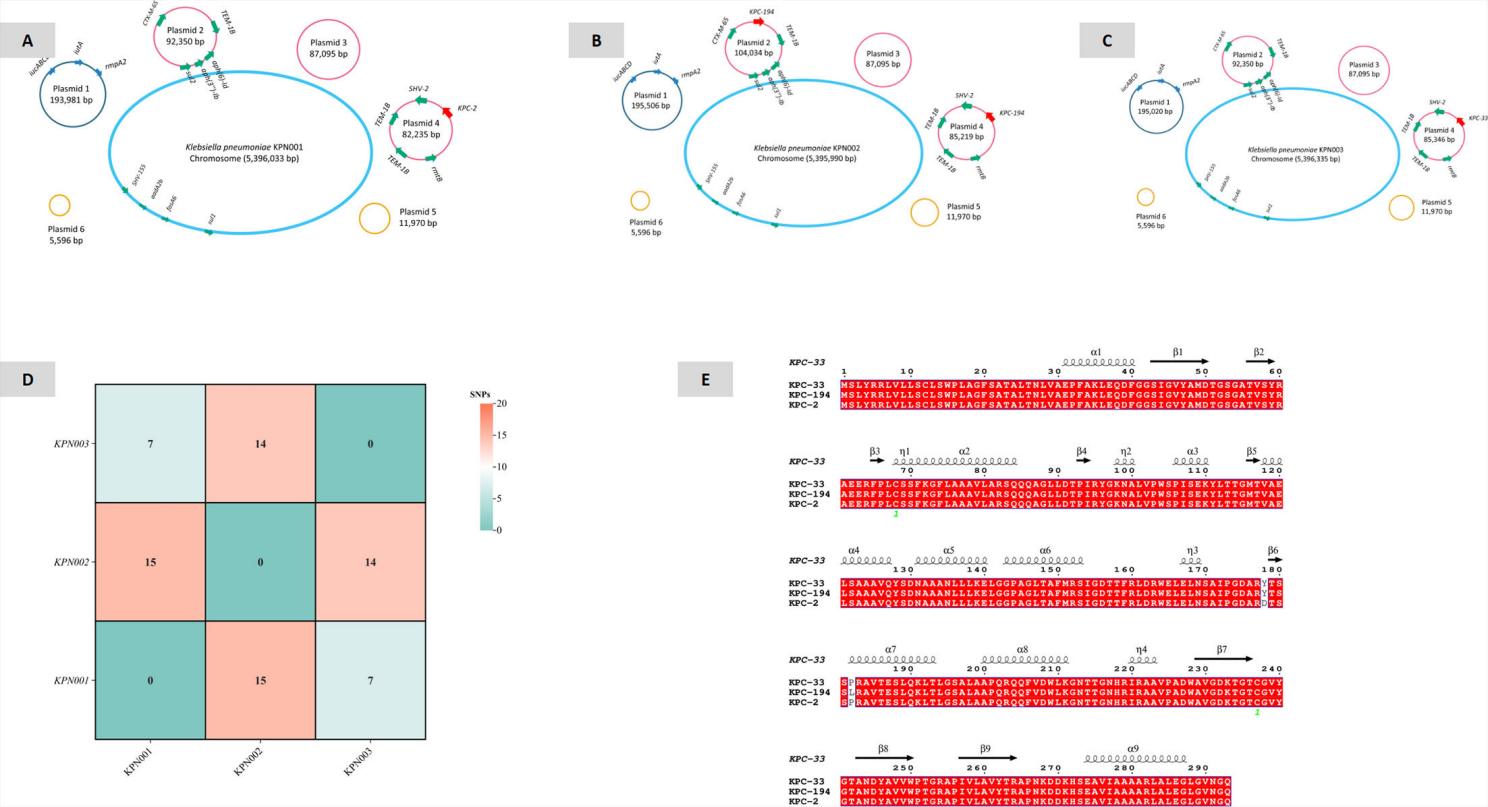
Distribution maps of resistance genes and virulence genes in *K. pneumoniae* KPN001 and KPN002. (**A**) Distribution map of resistance and virulence genes in *K. pneumoniae* KPN001. (**B**) Distribution map of resistance and virulence genes in *K. pneumoniae* KPN002. (**C**) Distribution map of resistance and virulence genes in *K. pneumoniae* KPN003. (**D**) Number of single-nucleotide polymorphisms between *K. pneumoniae* KPN001, KPN002, and KPN003. (**E**) Amino acid mutation sites of KPC variants, compared with KPC-2.

The patient was a 94-year-old male who was admitted to the hospital in September 2022 due to sepsis, severe pneumonia, and other conditions. During hospitalization, CRKP was isolated from the patient’s sputum sample in September 2022 (the day on which CRKP was detected in the patient was designated as day 1). AST results showed that this strain was susceptible to ceftazidime-avibactam. The patient then received intravenous infusion of ceftazidime-avibactam at a dose of 2.5 g every 8 hours for a 14-day course. One month later, CRKP (ceftazidime-avibactam-susceptible) was re-isolated from the patient’s sputum (designated as *K. pneumoniae* KPN001) and blood culture. The APB/EDTA method indicated that this strain produced class A serine carbapenemases, and the NG-Test Carba-5 showed a positive result for KPC; subsequent sequencing revealed that the strain carried the *bla*_KPC-2_ gene. The patient received ceftazidime-avibactam treatment again at a dose of 2.5 g every 8 hours. Subsequently, ceftazidime-avibactam-resistant CRKP strains were successively isolated from the patient’s blood culture and sputum (designated as *K. pneumoniae* KPN002 and *K. pneumoniae* KPN003, respectively). Then, whole-genome sequencing (WGS) showed that *K. pneumoniae* KPN002 carried *bla*_KPC-194_, and *K. pneumoniae* KPN003 carried *bla*_KPC-33_. Eventually, the patient died due to bacterial pneumonia, sepsis, septic shock, and other causes (Fig. 1 and 2A through C).

### Whole-genome sequencing results

WGS results showed that all three *K. pneumoniae* strains, KPN001, KPN002, and KPN003, belonged to the ST11-KL64-O2a type. Pairwise comparisons among the three strains revealed that their single-nucleotide polymorphism differences were all within 15, indicating that they belong to the exact clone (Fig. 2D). *K. pneumoniae* KPN001 carried the *bla*_KPC-2_, while *K. pneumoniae* KPN002 carried *bla*_KPC-194_ (NG_242186), and *K. pneumoniae* KPN003 carried *bla*_KPC-33_. KPC-33 had one amino acid site mutation compared with KPC-2, namely, D179Y. KPC-194 had two amino acid site mutations compared with KPC-2, specifically D179Y and P183L (Fig. 2E). All three *K. pneumoniae* strains carried the same resistance genes, including extended-spectrum β-lactamase genes (*bla*_SHV-12_, *bla*_LAP-2_, *bla*_CTX-M-65_, and *bla*_TEM-1B_), carbapenemase genes (KPN001, *bla*_KPC-2_; KPN002, *bla*_KPC-194_; and KPN003, *bla*_KPC-33_), *qnrS1*, *fosA6*, *dfrA14*, *tet(A)*, *sul1*, *sul2*, *catA2*, *addA2b*, *rmtB*, *aph(6)-Id*, *aph(3′)-Ib*, and *mph(A)*.

### KPC mutations mediate resistance of *K. pneumoniae* to ceftazidime-avibactam

BMD results showed that *K. pneumoniae* KPN001 was resistant to β-lactam antimicrobials (including imipenem, meropenem, ceftazidime, and cefepime), traditional β-lactam/β-lactamase inhibitor combinations (including piperacillin-tazobactam and cefoperazone-sulbactam), amikacin, and ciprofloxacin, intermediate to meropenem-vaborbactam, but susceptible to ceftazidime-avibactam, aztreonam-avibactam, imipenem-relebactam, colistin, tigecycline, and eravacycline. *K. pneumoniae* KPN002 and KPN003 were resistant to most β-lactam antimicrobials (including ceftazidime and cefepime), piperacillin-tazobactam, cefoperazone-sulbactam, ceftazidime-avibactam, as well as amikacin and ciprofloxacin, but susceptible to imipenem, aztreonam-avibactam, imipenem-relebactam, and meropenem-vaborbactam, and intermediate to meropenem. Cefiderocol was susceptible to all three test strains; however, the *bla*_KPC-Variant_-positive strains exhibited a four- to eightfold increase in MIC compared with the *bla*_KPC-2_-positive strain (Table 1).

**TABLE 1:** Antimicrobial susceptibility of clinical isolates, transformants, and clonal strains of *Klebsiella pneumoniae^a^*

Antimicrobial agents	Clinical strains’ MIC (μg/mL)			Conjugation (μg/mL)				Quality control strains (μg/mL) (QC range)	
	KPN001 (*bla*_KPC-2_)	KPN002 (*bla*_KPC-194_)	KPN003 (*bla*_KPC-33_)	*bla*_KPC-2_-EC600	*bla*_KPC-194_-EC600	*bla*_KPC-33_-EC600	EC600	*E. coli* ATCC 25922	*K. pneumoniae* BAA-1705
Imipenem	128	0.5	1	2	0.25	0.25	0.25	0.06 (0.06–0.5)	16 (4–16)
Meropenem	>64	2	2	4	0.06	0.06	≤0.03	≤0.03 (0.008–0.06)	32 (8–64)
Imipenem-relebactam	0.5	1	0.25	0.25	0.19	0.25	0.19	0.125 (0.06–0.25)	0.032 (0.03–0.25)
Meropenem-vaborbactam	8	2	2	≤0.03	≤0.03	≤0.03	≤0.03	≤0.03 (0.008–0.06)	≤0.03 (0.008–0.06)
Ceftazidime-avibactam	4	>64	>64	0.25	64	32	0.25	0.06 (0.06–0.5)	0.5 (0.25–2)
Aztreonam-avibactam	1	2	2	0.25	0.5	0.125	≤0.03	0.03 (0.03–0.12)	–
Cefiderocol	0.5	2	4	0.06	0.094	0.5	≤0.016	0.125 (0.06–0.5)	–
Cefepime	>32	>32	>32	16	16	16	≤0.25	≤0.25 (0.03–0.12)	–
Ceftazidime	>32	>32	>32	>32	>32	>32	0.5	0.25 (0.06–0.5)	–
Ceftriaxone	>32	>32	>32	>32	>32	>32	≤0.25	≤0.25 (0.03–0.12)	–
Aztreonam	>128	>128	>128	>128	>128	>128	≤1	≤1 (0.06–0.5)	–
Cefoperazone-sulbactam	>128	>128	>128	64	32	32	≤1	–	–
Piperacillin-tazobactam	>256	>256	>256	256	64	8	≤2	≤2 (1–8)	–
Eravacycline	1	1	1	0.25	0.125	0.125	0.125	0.06 (0.016–0.12)	–
Amikacin	>128	>128	>128	>128	>128	≤1	≤1	1 (0.25–4)	–
Ciprofloxacin	>8	>8	>8	4	4	4	0.25	0.008 (0.004–0.016)	–
Tigecycline	1	1	1	0.5	0.25	0.25	0.125	0.125 (0.02–0.25)	–
Colistin	0.25	0.25	0.25	0.25	≤0.125	0.25	≤0.125	–	–
Sulfamethoxazole-trimethoprim	>16	>16	>16	>16	>16	>16	≤0.25	≤0.25 (≤0.5)	–

^*a*^ “–” indicates that there is no MIC QC range for this strain in CLSI M100.

Conjugation results showed that, compared with *E. coli* EC600, the MICs of ceftazidime-avibactam, aztreonam-avibactam, cefepime, ceftazidime, ceftriaxone, aztreonam, cefoperazone-sulbactam, piperacillin-tazobactam, amikacin, ciprofloxacin, and trimethoprim-sulfamethoxazole against *E. coli* EC600 carrying the *bla*_KPC-194_ plasmid increased by 256-, 64-, 64-, 64-, 128-, 128-, 32-, 32-, 32-, 16-, and 64-fold, respectively; however, the MICs of imipenem and meropenem remained essentially unchanged. Compared with *E. coli* EC600 carrying the *bla*_KPC-2_ plasmid, the MICs of ceftazidime-avibactam and aztreonam-avibactam against *E. coli* EC600 carrying the *bla*_KPC-194_ plasmid increased by 256-fold. In contrast, the MICs of imipenem, meropenem, and piperacillin-tazobactam decreased by 4-, 64-, and 4-fold, respectively. Compared with *E. coli* EC600 carrying the *bla*_KPC-33_ plasmid, the MICs of aztreonam-avibactam and piperacillin-tazobactam against *E. coli* EC600 carrying the *bla*_KPC-194_ plasmid increased by four- and eightfold, respectively. At the same time, there were no significant changes in the MICs of other antimicrobial agents (Table 1). The conjugation frequencies of *bla*_KPC_-positive transconjugants in KPN001, KPN002, and KPN003 were 2.9 × 10⁻⁶, 1.1 × 10⁻⁵, and 1.8 × 10⁻⁶, respectively.

Cloning assay results showed that, compared with the *E. coli* DH5α strain carrying pHSG398, the MIC of imipenem against *E. coli* DH5α-pHSG398-*bla*_KPC-194_ increased by at least twofold. The MICs of ceftazidime and ceftazidime-avibactam increased by 512- and 64-fold, respectively, while there were no significant changes in the MICs of other antimicrobial agents. Compared with *E. coli* DH5α (pHSG398-*bla*_KPC-2_), the MICs of imipenem, meropenem, cefepime, ceftriaxone, aztreonam, cefoperazone-sulbactam, and piperacillin-tazobactam against *E. coli* DH5α (pHSG398-*bla*_KPC-194_) decreased by 64-, 128-, 32-, 64-, 128-, 64-, and 32-fold or more, respectively, while the MIC of ceftazidime-avibactam increased by 128-fold. The *E. coli* DH5α strains carrying either pHSG398-*bla*_KPC-33_ or pHSG398-*bla*_KPC-194_ exhibited similar susceptibility profiles. Notably, both constructs mediated a reduced susceptibility to ceftazidime-avibactam, coupled with increased susceptibility to imipenem and meropenem (Table 2).

**TABLE 2:** Antimicrobial susceptibility of clonal strains of *Klebsiella pneumoniae*

Antimicrobial agents	Clonal strains (μg/mL)			
	*E. coli* DH5α pHSG398-*bla*_KPC-2_	*E. coli* DH5α pHSG398-*bla*_KPC-194_	*E. coli* DH5α pHSG398-*bla*_KPC-33_	*E. coli* DH5α pHSG398
Imipenem	8	0.125	0.125	≤0.06
Meropenem	8	≤0.06	≤0.06	≤0.06
Imipenem-relebactam	0.125	0.125	0.125	0.094
Meropenem-vaborbactam	≤0.06	≤0.06	≤0.06	≤0.06
Ceftazidime-avibactam	0.25	32	4	0.125
Aztreonam-avibactam	0.06	0.125	≤0.06	≤0.06
Cefiderocol	≤0.016	0.094	0.064	≤0.016
Cefepime	16	0.5	0.5	0.125
Ceftazidime	32	>32	4	≤0.06
Ceftriaxone	32	0.5	8	≤0.25
Aztreonam	128	≤1	≤1	≤1
Cefoperazone-sulbactam	64	≤1	≤1	≤1
Piperacillin-tazobactam	>256	8	4	4
Eravacycline	0.125	≤0.06	≤0.06	≤0.06
Amikacin	0.5	1	1	0.5
Ciprofloxacin	≤0.06	≤0.06	≤0.06	≤0.06
Tigecycline	0.25	0.125	0.125	≤0.06
Colistin	0.25	0.25	0.25	0.25
Sulfamethoxazole-trimethoprim	≤0.06	≤0.06	≤0.06	≤0.06

### Identification of plasmids carrying the *bla*_KPC_

**Fig 3:**
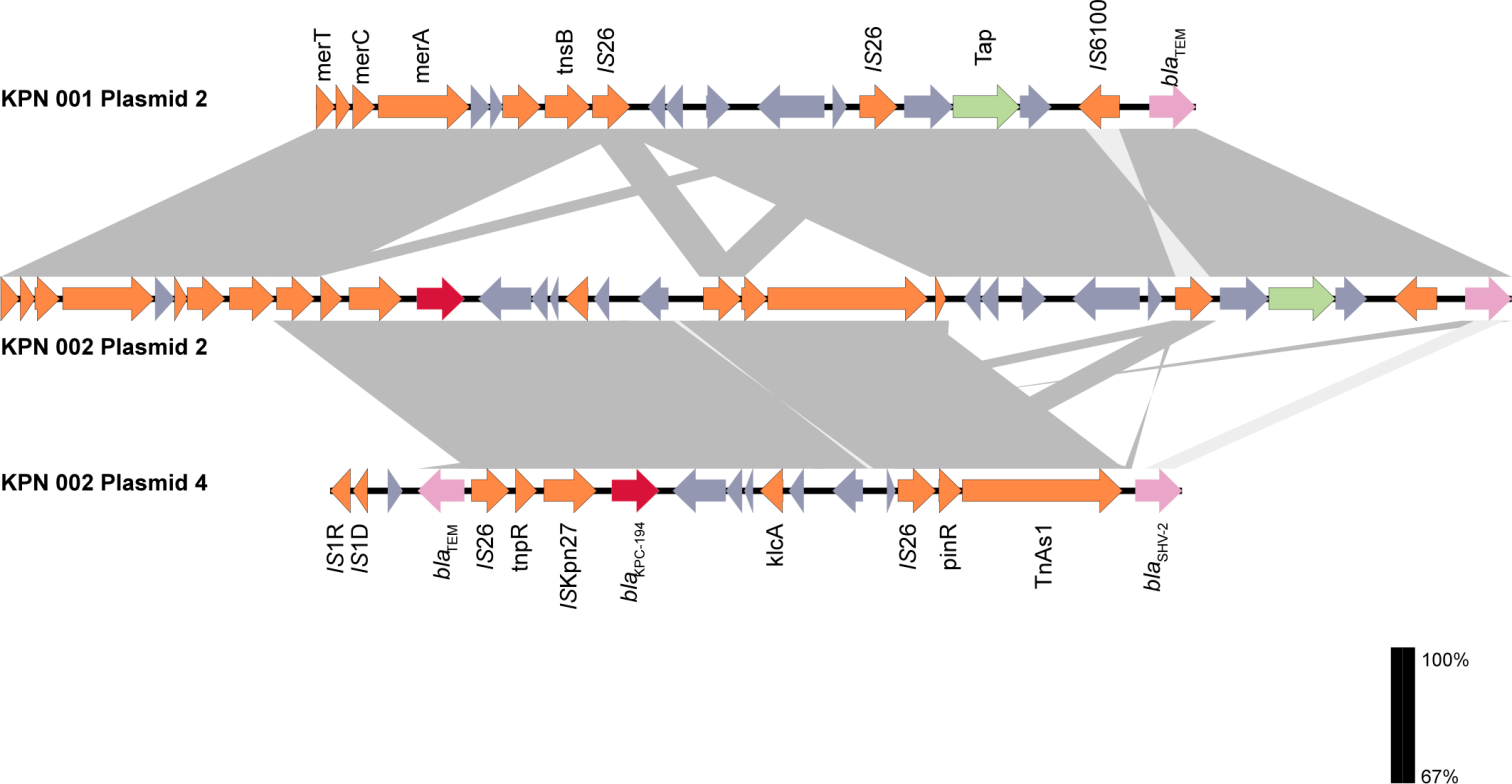
Genetic environment maps of *bla*_KPC-194_ in plasmid 2 and plasmid 4 of *K. pneumoniae* KPN002 and “relative genes” in plasmid 2 of *K. pneumoniae* KPN002.

*K. pneumoniae* KPN001 consists of one 5,396,033 bp chromosome and six plasmids, namely, plasmid 1 (193,981 bp), plasmid 2 (92,350 bp), plasmid 3 (87,095 bp), plasmid 4 (85,235 bp), plasmid 5 (11,970 bp), and plasmid 6 (5,596 bp) (Fig. 2A; Table 3). The *bla*_KPC-2_ is located on the 85,235 bp IncR-type plasmid 4, with a surrounding genetic structure of IS*26-TnpR_Tn3*-IS*KPn27-bla*_KPC-2_-IS*KPn6-KclA*-IS*26-pinR-Tn3_TnAs1*. This plasmid also carries *bla*_SHV-12_, two copies of *bla*_TEM-1B_, and *rmtB* (Fig. 3).

**TABLE 3:** Molecular characteristics of *Klebsiella pneumoniae* KPN001, KPN002, and their plasmids

Strains	Chromosome or plasmid (size)	Plasmid type	Resistance genes	Hypervirulent genes
KPN001	Chr (5,396,033 bp)	–^*a*^	*aadA2b*, *bla*_SHV-155_, *fosA6*, *sul1*	
	Plasmid 1 (193,981 bp)	IncFIB(K)/IncHI1B (pNDM-MAR)	–	*iucABCD*, *iutA*, *rmpA2*
	Plasmid 2 (92,350 bp)	IncFII(pHN7A8)	*bla*_CTX-M-65_, *bla*_TEM-1B_, *aph(6)-Id*, *aph(3″)-Ib*, *mph(A)*, *sul2*	–
	Plasmid 3 (87,095 bp)	IncFII(pSDP9R)	*bla*_LAP-2_, *catA2*, *tet(A*), *dfrA14*, *qnrS1*, *sul2*	–
	Plasmid 4 (85,235 bp)	IncR	*bla*_KPC-2_, *bla*_SHV-12_, *bla*_TEM-1B_ (7694..8554), *bla*_TEM-1B_ (51909..52769), *rmtB*	–
	Plasmid 5 (11,970 bp)	ColRNAI	–	–
	Plasmid 6 (5,596 bp)	–	–	–
KPN002	Chr (5,395,990 bp)	–	*aadA2b*, *bla*_SHV-155_, *fosA6*, *sul1*	–
	Plasmid 1 (195,506 bp)	IncFIB(K)/IncHI1B (pNDM-MAR)	–	*iucABCD*, *iutA*, *rmpA2*
	Plasmid 2 (104,034 bp)	IncFII(pHN7A8)	*bla*_KPC-194_, *bla*_CTX-M-65_, *bla*_TEM-1B_, *aph(6)-Id*, *aph(3″)-Ib*, *mph(A)*, *sul2*	–
	Plasmid 3 (87,095 bp)	IncFII(pSDP9R)	*bla*_LAP-2_, *catA2*, *tet(A*), *dfrA14*, *qnrS1*, *sul2*	–
	Plasmid 4 (85,219 bp)	IncR	*bla*_KPC-194_, *bla*_SHV-12_, *bla*_TEM-1B_ (7694..8554), *bla*_TEM-1B_ (51909..52769), *rmtB*	–
	Plasmid 5 (11,970 bp)	ColRNAI	–	–
	Plasmid 6 (5,596 bp)	–	–	–
KPN003	Chr (5,396,335 bp)	–	*aadA2b*, *bla*_SHV-155_, *fosA6*, *sul1*	
	Plasmid 1 (195,020 bp)	IncFIB(K)/IncHI1B (pNDM-MAR)	–	*iucABCD*, *iutA*, *rmpA2*
	Plasmid 2 (92,350 bp)	IncFII(pHN7A8)	*bla*_CTX-M-65_, *bla*_TEM-1B_, *aph(6)-Id*, *aph(3″)-Ib*, *mph(A)*, *sul2*	–
	Plasmid 3 (87,095 bp)	IncFII(pSDP9R)	*bla*_LAP-2_, *catA2*, *tet(A*), *dfrA14*, *qnrS1*, *sul2*	–
	Plasmid 4 (85,346 bp)	IncR	*bla*_KPC-33_, *bla*_SHV-12_, *bla*_TEM-1B_ (7694..8554), *bla*_TEM-1B_ (51909..52769), *rmtB*	–
	Plasmid 5 (11,970 bp)	ColRNAI	–	–
	Plasmid 6 (5,596 bp)	–	–	–

^*a*^ “–” denotes a negative result, indicating the absence of the relevant gene or that the plasmid type is unknown.

**Fig 4:**
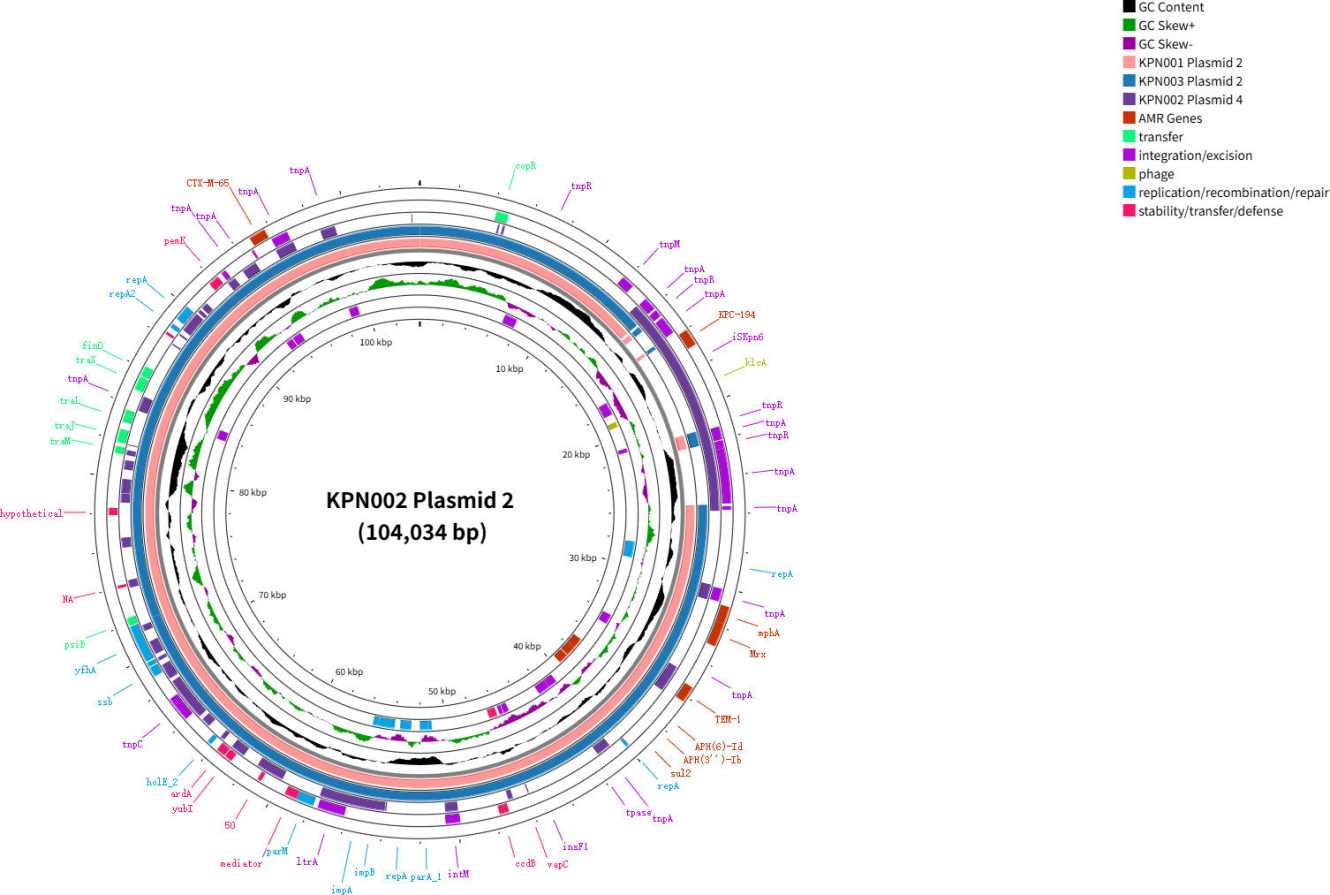
Comparison of KPN002 plasmid 2, KPN001 plasmid 2, KPN003 plasmid 2, and KPN002 plasmid 4 using Proksee.

**Fig 5:**
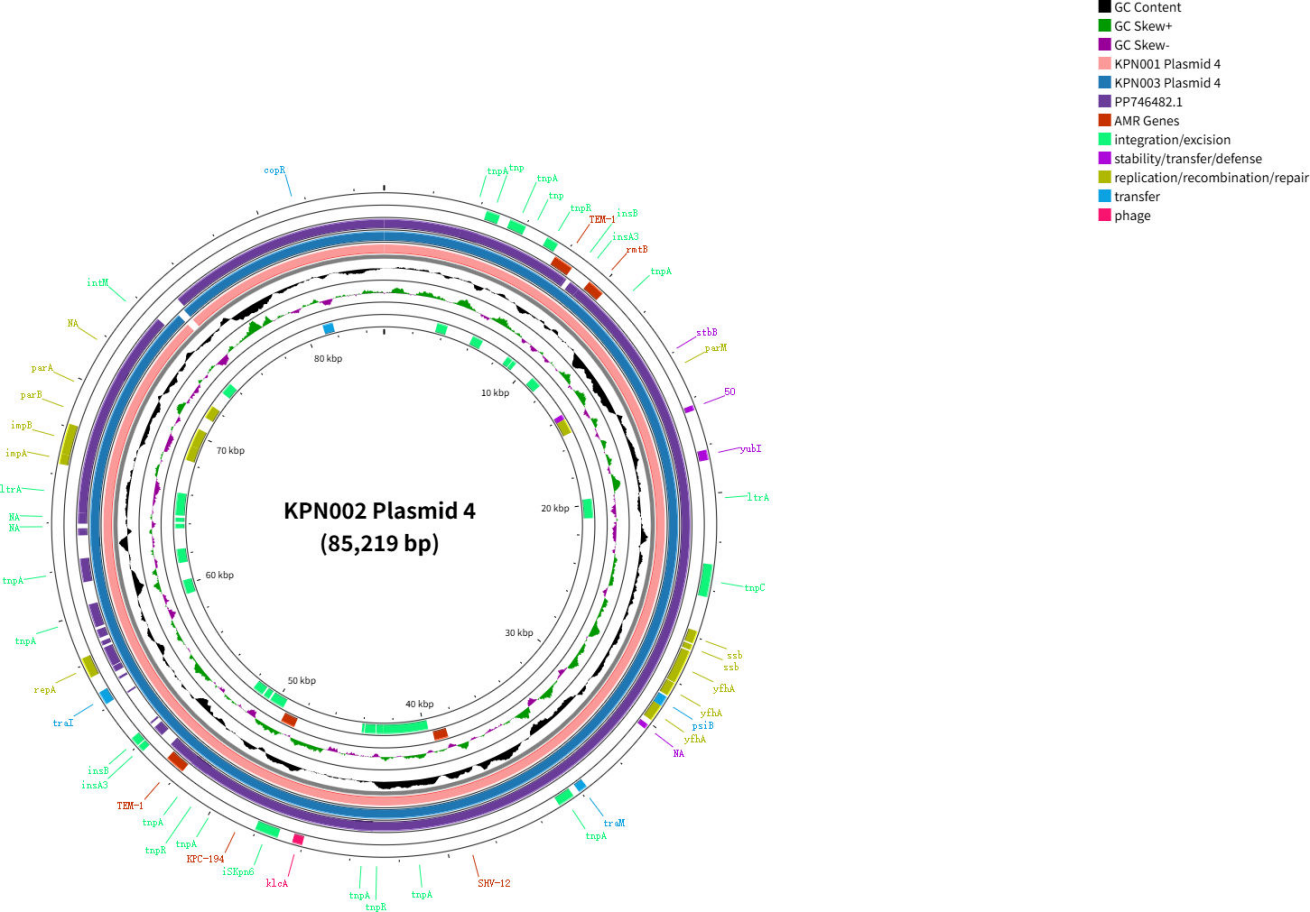
Comparison of KPN002 plasmid 4, KPN001 plasmid 4, KPN003 plasmid 4, and pKPC-2SCNJ51 using Proksee. BLAST search of the sequence in GenBank revealed that the sequence of plasmid 4 shared similarity with that of the pKPC-2SCNJ51 plasmid (135,683 bp; GenBank accession no. PP746482.1), which was isolated from Sichuan Province, China, in 2024, with 89% coverage and 100% identity.

*K. pneumoniae* KPN002 comprises one 5,395,990 bp chromosome and six plasmids: plasmid 1 (195,506 bp), plasmid 2 (104,034 bp), plasmid 3 (87,095 bp), plasmid 4 (85,219 bp), plasmid 5 (11,970 bp), and plasmid 6 (5,596 bp) (Fig. 2B; Table 3). In KPN002, the *bla*_KPC-194_ is present on both the 104,034 bp IncFII-type plasmid 2 and the 85,219 bp IncR-type plasmid 4 (Fig. 4 and 5). These two *bla*_KPC-194_-carrying plasmids (plasmid 2 and plasmid 4) have an identical complete genetic structure of IS*26-TnpR_Tn3*-IS*KPn27-bla*_KPC-194_-IS*KPn6-KclA-IS26-pinR-Tn3_TnAs1*, with a length of 12,152 bp (Fig. 3).

*K. pneumoniae* KPN003 consists of one 5,396,335 bp chromosome and six plasmids, namely, plasmid 1 (195,020 bp), plasmid 2 (92,350 bp), plasmid 3 (87,095 bp), plasmid 4 (85,346 bp), plasmid 5 (11,970 bp), and plasmid 6 (5,596 bp) (Fig. 2C; Table 3). The *bla*_KPC-33_ is located on the 85,346 bp IncR-type plasmid 4, with a surrounding genetic structure of IS*26-TnpR_Tn3*-IS*KPn27-bla*_KPC-2_-IS*KPn6-KclA*-IS*26-pinR-Tn3_TnAs1*. This plasmid also carries *bla*_SHV-12_, two copies of *bla*_TEM-1B_, and *rmtB* (Fig. 5).

The total of three strains shared identical plasmids 3, 5, and 6. Plasmids 2 of *K. pneumoniae* KPN003 share 100% similarity with *K. pneumoniae* KPN001. Compared with plasmid 2 (92,350 bp) of KPN001 and KPN003, plasmid 2 (104,034 bp) of KPN002 contains an additional 11,684 bp fragment, designated as ΔIS26-3′-*TnpR_Tn3*-IS*KPn27-bla*_KPC-194_-IS*KPn6-KclA-IS26-pinR-Tn3_TnAs1*.

### Identification of hypervirulent IncHI1B/repB-type plasmids

The total of three strains carries an IncFIB(K)/IncHI1B (pNDM- MAR)-type plasmid. This plasmid contains the same virulence genes, including *iucABCD* and *iutA*, as well as *rmpA2*; no *rmpA* or *iroBCDN* genes were detected. This plasmid shares 100% coverage and 99.91% similarity with pCRKP-35_Vir (accession no. CP102638) in the NCBI database, which was isolated from Zhejiang, China, in 2023.

## DISCUSSION

Ceftazidime-avibactam is a crucial agent for the treatment of infections caused by CRKP-producing KPC or OXA-48-like carbapenemase. However, with the clinical application of this class of drugs, strains resistant to it have been continuously emerging (14, 15). Currently, the primary mechanism of ceftazidime-avibactam resistance in KPC-producing CRKP is mutations in the *bla*_KPC_ gene (16). In this study, a novel KPC variant (KPC-194) was successfully isolated and identified. Functional studies on this novel KPC variant revealed that it can mediate bacterial resistance to ceftazidime-avibactam while restoring susceptibility to imipenem and meropenem. Studies have shown that the number of KPC-variant-producing bacteria is currently increasing (14, 17, 18). Moreover, the host bacteria are no longer limited to *K. pneumoniae*. Cases of KPC mutations have been reported in *E. coli*, *Serratia marcescens*, and *Pseudomonas aeruginosa*, among others, which has incredibly aroused public concern about infections caused by this highly drug-resistant bacterium (19, 20). Similar to previous studies, a history of ceftazidime-avibactam treatment is the main trigger for *bla*_KPC_ mutations. In this study, during infection with KPC-2-producing *K. pneumoniae*, the patient received ceftazidime-avibactam for anti-infective therapy. Subsequently, KPC-194-producing *K. pneumoniae* and KPC-33-producing *K. pneumoniae* were detected in both the patient’s blood culture and sputum, respectively.

Clinically, the phenomenon of bacterial resistance to ceftazidime-avibactam mediated by KPC mutations can be traced back to 2016. Since then, the number of international reports on KPC variants has increased significantly. Studies have shown that blaKPC mutation hotspots are concentrated in three regions, namely, the Ω-loop (positions 164–179), loops 237–243, and loops 266–275 (21). In Europe and the Americas, KPC-31 (D179Y, a mutation based on KPC-3) is the predominant variant. In contrast, KPC-33 (D179Y) is the most commonly reported KPC variant and the earliest KPC variant reported in China (15, 22). Relative to KPC-2, KPC-194 exhibits two amino acid substitutions: D179Y and P183L. KPC-194-producing *K. pneumoniae* exhibits similar antimicrobial susceptibility profiles to those of KPC-33-producing strains (13). Similarly, KPC-194 cannot be effectively detected by either the APB/EDTA method or the NG-Test Carba 5 assay, resulting in false-negative results (15). Compared with the KPC-2-carrying clone, cloning assay results of the KPC-194-carrying clone demonstrated increased resistance to ceftazidime and ceftazidime-avibactam. In contrast, its susceptibility to other β-lactam agents was significantly enhanced, including aztreonam, cefepime, piperacillin-tazobactam, imipenem, and meropenem.

Two genetic elements, the Tn4401 transposon and the Tn3-Tn4401 transposon chimera, represent the primary genetic contexts for *bla*_KPC_ (23). In China, the genetic context of *bla*_KPC_ is distinctive, with the Tn*1721-bla*_KPC_-IS*26* transposon being the most prevalent (24). However, in this study, *bla*_KPC-194_ was carried simultaneously by a 104,034 bp IncFII(pHN7A8) plasmid and an 85,219 bp IncR plasmid, both of which shared the same genetic structure: IS*26-TnpR_Tn3*-IS*KPn27-bla*_KPC-194_-IS*KPn6-KclA-IS26-pinR-Tn3_TnAs1*. During the *K. pneumoniae* infection, *bla*_KPC-194_ may have undergone horizontal transfer from the IncR plasmid (85,219 bp) to the IncFII(pHN7A8) plasmid (104,034 bp) via an IS*26*-mediated replicative transposition event. However, it is interesting to note that although *bla*_KPC-194_ is located on two plasmids in KPN002, its resistance phenotype does not show a significant difference compared to that of a *Klebsiella pneumoniae* strain carrying a single copy of *bla*_KPC-33_. Meanwhile, the *bla*_KPC_-harboring elements in these strains exhibited low conjugation frequencies (on the order of 10⁻⁶ to 10⁻⁵), suggesting a limited horizontal transfer potential under laboratory conditions. Nevertheless, the successful acquisition of transconjugants confirms that this IncR plasmid possesses the potential for cross-strain transfer in the presence of helper plasmids.

Since the first detection of hypervirulent *K. pneumoniae* (HvKP) in patients with liver abscesses in Taiwan, China, in the 1980s, the prevalence of HvKP has been increasing globally (25). Concurrently, CRKP has acquired hypervirulence plasmids, enabling the bacteria to exhibit both hypervirulence and high antibiotic resistance, an issue that has become a significant threat to global public health. Owing to the influence of the thick capsular polysaccharide and other factors, it is less complicated for CRKP to capture virulence plasmids than for HvKP to capture highly resistant plasmids. Hv-CRKP was first identified in 2015 (26). Since then, ST11-KL64 hv-CRKP has emerged as the most prevalent hv-CRKP clone in clinical settings across China (27). Consistent with our previous study (28), we also identified the presence of a large hypervirulence plasmid of the IncFIB(K)/IncHI1B type in the *bla*_KPC-135_-ST11-KL64 hv-CRKP strain. The hypervirulence plasmid and the resistance plasmid (the IncFII/IncR plasmid carrying *bla*_KPC-194_) co-exist within the host, forming a typical “hypervirulence–high resistance” complex. Similar to the jumping and dissemination of resistance genes between plasmids, the stable maintenance and transmission of such hypervirulence plasmids collectively exacerbate the clinical threat posed by ST11-KL64 hv-CRKP and increase the difficulty of its prevention and control.

## Data Availability

The data used during the current study are available from the corresponding author on reasonable request. The bacterial genome sequences have been uploaded to NCBI with the following BioSample accession nos.: SAMN54245612, SAMN54245613, and SAMN54245614.

## References

[1] Potter RF, D’Souza AW, Dantas G. 2016. The rapid spread of carbapenem-resistant Enterobacteriaceae. Drug Resist Updat 29:30–46. doi:10.1016/j.drup.2016.09.002 27912842 PMC5140036

[2] van Duin D, Doi Y. 2017. The global epidemiology of carbapenemase-producing Enterobacteriaceae. Virulence 8:460–469. doi:10.1080/21505594.2016.1222343 27593176 PMC5477705

[3] Wang Q, Wang R, Wang S, Zhang A, Duan Q, Sun S, Jin L, Wang X, Zhang Y, Wang C, Kang H, Zhang Z, Liao K, Guo Y, Jin L, Liu Z, Yang C, Wang H, China Carbapenem-Resistant Enterobacterales (CRE) Network. 2024. Expansion and transmission dynamics of high risk carbapenem-resistant Klebsiella pneumoniae subclones in China: an epidemiological, spatial, genomic analysis. Drug Resist Updat 74:101083. doi:10.1016/j.drup.2024.101083 38593500

[4] Wang M, Earley M, Chen L, Hanson BM, Yu Y, Liu Z, Salcedo S, Cober E, Li L, Kanj SS, et al.. 2022. Clinical outcomes and bacterial characteristics of carbapenem-resistant Klebsiella pneumoniae complex among patients from different global regions (CRACKLE-2): a prospective, multicentre, cohort study. Lancet Infect Dis 22:401–412. doi:10.1016/S1473-3099(21)00399-6 34767753 PMC8882129

[5] Han R, Shi Q, Wu S, Yin D, Peng M, Dong D, Zheng Y, Guo Y, Zhang R, Hu F, China Antimicrobial Surveillance Network (CHINET) Study Group. 2020. Dissemination of carbapenemases (KPC, NDM, OXA-48, IMP, and VIM) among carbapenem-resistant Enterobacteriaceae isolated from adult and children patients in China. Front Cell Infect Microbiol 10:314. doi:10.3389/fcimb.2020.00314 32719751 PMC7347961

[6] Tamma PD, Heil EL, Justo JA, Mathers AJ, Satlin MJ, Bonomo RA. 2024. Infectious diseases society of America 2024 guidance on the treatment of antimicrobial-resistant gram-negative infections. Clin Infect Dis:ciae403. doi:10.1093/cid/ciae403 39108079

[7] Tamma PD, Aitken SL, Bonomo RA, Mathers AJ, van Duin D, Clancy CJ. 2023. Infectious diseases society of America 2023 guidance on the treatment of antimicrobial resistant gram-negative infections. Clin Infect Dis:ciad428. doi:10.1093/cid/ciad428 37463564

[8] Ye H, Liu R, Shen J, Yang W, Hu T, Liu X, Wang K, Gong L, Xu H, Zhu J, Zheng Z, Zheng B. 2025. Unravelling tandem repeat-mediated mutagenesis drive rapid diversification of KPC enzymes: emergence of bla_KPC-263_ and enhanced resistance to ceftazidime-avibactam. EBioMedicine 121:105979. doi:10.1016/j.ebiom.2025.105979 41135172 PMC12593533

[9] Guo T, Ding L, Shen S, Tang C, Jiang T, Hu F. 2025. Molecular characterisation of two novel KPC variants mediating ceftazidime-avibactam resistance in ST11 Klebsiella pneumoniae. Int J Antimicrob Agents 66:107642. doi:10.1016/j.ijantimicag.2025.107642 41072859

[10] Wayne P. 2024. Performance standards for antimicrobial susceptibility testing. 34th ed. Clinical and Laboratory Standards Institute.

[11] Ding L, Shi Q, Han R, Yin D, Wu S, Yang Y, et al.. 2021. Comparison of four carbapenemase detection methods for bla(KPC-2) variants. Microbiol Spectr 9:e0095421. doi:10.1128/Spectrum.00954-21 34935416 PMC8693920

[12] Sullivan MJ, Petty NK, Beatson SA. 2011. Easyfig: a genome comparison visualizer. Bioinformatics 27:1009–1010. doi:10.1093/bioinformatics/btr039 21278367 PMC3065679

[13] Huang X, Shen S, Chang F, Liu X, Yue J, Xie N, Yin L, Hu F, Xiao D. 2023. Emergence of KPC-134, a KPC-2 variant associated with ceftazidime-avibactam resistance in a ST11 Klebsiella pneumoniae clinical strain. Microbiol Spectr 11:e0072523. doi:10.1128/spectrum.00725-23 37772834 PMC10580995

[14] Shields RK, Chen L, Cheng S, Chavda KD, Press EG, Snyder A, Pandey R, Doi Y, Kreiswirth BN, Nguyen MH, Clancy CJ. 2017. Emergence of ceftazidime-avibactam resistance due to plasmid-borne bla _KPC-3_ mutations during treatment of carbapenem-resistant Klebsiella pneumoniae infections. Antimicrob Agents Chemother 61:e02097-16. doi:10.1128/AAC.02097-16 28031201 PMC5328542

[15] Shi Q, Yin D, Han R, Guo Y, Zheng Y, Wu S, Yang Y, Li S, Zhang R, Hu F. 2020. Emergence and recovery of ceftazidime-avibactam resistance in blaKPC-33-Harboring Klebsiella pneumoniae sequence type 11 isolates in China. Clin Infect Dis 71:S436–S439. doi:10.1093/cid/ciaa1521 33367577

[16] Ding L, Shen S, Chen J, Tian Z, Shi Q, Han R, Guo Y, Hu F. 2023. Klebsiella pneumoniae carbapenemase variants: the new threat to global public health. Clin Microbiol Rev 36:e00008-23. doi:10.1128/cmr.00008-23 37937997 PMC10732083

[17] Vásquez-Ponce F, Bispo J, Becerra J, Fontana H, Pariona JGM, Esposito F, Fuga B, Oliveira FA, Brunetti F, Power P, Gutkind G, Schreiber AZ, Lincopan N. 2023. Emergence of KPC-113 and KPC-114 variants in ceftazidime-avibactam-resistant Klebsiella pneumoniae belonging to high-risk clones ST11 and ST16 in South America. Microbiol Spectr 11:e00374-23. doi:10.1128/spectrum.00374-23 37671877 PMC10580961

[18] De la Cadena E, Mojica MF, Rojas LJ, Castro BE, García-Betancur JC, Marshall SH, Restrepo N, Castro-Caro NP, Fonseca-Carrillo M, Pallares C, Bonomo RA, Villegas MV. 2024. First report of KPC variants conferring ceftazidime-avibactam resistance in Colombia: introducing KPC-197. Microbiol Spectr 12:e0410523. doi:10.1128/spectrum.04105-23 38700337 PMC11237465

[19] Faccone D, Mendieta JM, Albornoz E, Chavez M, Genero F, Echegorry M, et al.. 2022. Emergence of KPC-31, a KPC-3 variant associated with ceftazidime-avibactam resistance. Antimicrob Agents Chemother 66. doi:10.1128/aac.00648-22 PMC9664854 36286541

[20] Tu Y, Wang D, Zhu Y, Li J, Jiang Y, Wu W, Li X, Zhou H. 2022. Emergence of a KPC-90 Variant that confers resistance to ceftazidime-avibactam in an st463 carbapenem-resistant Pseudomonas aeruginosa strain. Microbiol Spectr 10:e0186921. doi:10.1128/spectrum.01869-21 35019766 PMC8754116

[21] Hobson CA, Pierrat G, Tenaillon O, Bonacorsi S, Bercot B, Jaouen E, Jacquier H, Birgy A. 2022. Klebsiella pneumoniae carbapenemase variants resistant to ceftazidime-avibactam: an evolutionary overview. Antimicrob Agents Chemother 66:e0044722. doi:10.1128/aac.00447-22 35980232 PMC9487638

[22] Ding L, Shen S, Han R, Yin D, Guo Y, Hu F. 2022. Ceftazidime-avibactam in combination with imipenem as salvage therapy for ST11 KPC-33-producing Klebsiella pneumoniae Antibiotics (Basel) 11:604. doi:10.3390/antibiotics11050604 35625247 PMC9138154

[23] Naas T, Cuzon G, Villegas MV, Lartigue MF, Quinn JP, Nordmann P. 2008. Genetic structures at the origin of acquisition of the beta-lactamase bla KPC gene. Antimicrob Agents Chemother 52:1257–1263. doi:10.1128/AAC.01451-07 18227185 PMC2292522

[24] Tang Y, Li G, Shen P, Zhang Y, Jiang X. 2022. Replicative transposition contributes to the evolution and dissemination of KPC-2-producing plasmid in Enterobacterales. Emerg Microbes Infect 11:113–122. doi:10.1080/22221751.2021.2013105 34846275 PMC8725868

[25] Fung C-P, Chang F-Y, Lee S-C, Hu B-S, Kuo BI-T, Liu C-Y, Ho M, Siu LK. 2002. A global emerging disease of Klebsiella pneumoniae liver abscess: is serotype K1 an important factor for complicated endophthalmitis? Gut 50:420–424. doi:10.1136/gut.50.3.420 11839725 PMC1773126

[26] Yao B, Xiao X, Wang F, Zhou L, Zhang X, Zhang J. 2015. Clinical and molecular characteristics of multi-clone carbapenem-resistant hypervirulent (hypermucoviscous) Klebsiella pneumoniae isolates in a tertiary hospital in Beijing, China. Int J Infect Dis 37:107–112. doi:10.1016/j.ijid.2015.06.023 26141415

[27] Jia X, Zhu Y, Jia P, Li C, Chu X, Sun T, Liu X, Yu W, Chen F, Xu Y, Yang Q. 2024. The key role of iroBCDN-lacking pLVPK-like plasmid in the evolution of the most prevalent hypervirulent carbapenem-resistant ST11-KL64 Klebsiella pneumoniae in China. Drug Resist Updat 77:101137. doi:10.1016/j.drup.2024.101137 39178714

[28] Shi Q, Shen S, Tang C, Ding L, Guo Y, Yang Y, Wu S, Han R, Yin D, Hu F. 2024. Molecular mechanisms responsible KPC-135-mediated resistance to ceftazidime-avibactam in ST11-K47 hypervirulent Klebsiella pneumoniae. Emerg Microbes Infect 13:2361007. doi:10.1080/22221751.2024.2361007 38801099 PMC11172257

